# CABYR isoforms expressed in late steps of spermiogenesis bind with AKAPs and ropporin in mouse sperm fibrous sheath

**DOI:** 10.1186/1477-7827-8-101

**Published:** 2010-08-23

**Authors:** Yan-Feng Li, Wei He, Young-Hwan Kim, Arabinda Mandal, Laura Digilio, Ken Klotz, Charles J Flickinger, John C Herr

**Affiliations:** 1 Department of Urology, Daping Hospital, Institute of Surgery Research, Third Military Medical University, Chongqing 400042, PR China; 2 Department of Obstetrics and Gynecology, Southwest Hospital, Third Military Medical University, Chongqing 400038, PR China; 3 Center for Research in Contraceptive and Reproductive Health, Department of Cell Biology, University of Virginia, P.O. Box 800732, Charlottesville, VA 22908, USA

## Abstract

**Background:**

CABYR is a polymorphic calcium-binding protein of the sperm fibrous sheath (FS) which gene contains two coding regions (CR-A and CR-B) and is tyrosine as well as serine/threonine phosphorylated during in vitro sperm capacitation. Thus far, the detailed information on CABYR protein expression in mouse spermatogenesis is lacking. Moreover, because of the complexity of this polymorphic protein, there are no data on how CABYR isoforms associate and assemble into the FS.

**Methods:**

The capacity of mouse CABYR isoforms to associate into dimers and oligomers, and the relationships between CABYR and other FS proteins were studied by gel electrophoresis, Western blotting, immunofluorescence, immunoprecipitation and yeast two-hybrid analyses.

**Results:**

The predominant form of mouse CABYR in the FS is an 80 kDa variant that contains only CABYR-A encoded by coding region A. CABYR isoforms form dimers by combining the 80 kDa CABYR-A-only variant with the 50 kDa variant that contains both CABYR-A and CABYR-B encoded by full length or truncated coding region A and B. It is proposed that this step is followed by the formation of larger oligomers, which then participate in the formation of the supramolecular structure of the FS in mouse sperm. The initial expression of CABYR occurs in the cytoplasm of spermatids at step 11 of spermiogenesis and increases progressively during steps 12-15. CABYR protein gradually migrates into the sperm flagellum and localizes to the FS of the principal piece during steps 15-16. Deletion of the CABYR RII domain abolished the interaction between CABYR and AKAP3/AKAP4 but did not abolish the interaction between CABYR and ropporin suggesting that CABYR binds to AKAP3/AKAP4 by its RII domain but binds to ropporin through another as yet undefined region.

**Conclusions:**

CABYR expresses at the late stage of spermiogenesis and its isoforms oligomerize and bind with AKAPs and ropporin. These interactions strongly suggest that CABYR participates in the assembly of complexes in the FS, which may be related to calcium signaling.

## Background

The fibrous sheath (FS), a unique cytoskeletal structure specific to the sperm, surrounds the axoneme and outer dense fibers and consists of two longitudinal columns connected by closely arrayed semicircular ribs. The FS is located only in the principal piece, a region devoid of mitochondria, and it assembles in a distal to proximal direction during spermiogenesis. The FS has been proposed to function as a protective girdle for the axoneme [1,2], influence the degree of flexibility, plane of flagellar motion, the shape of the flagellar beat and as a scaffold for enzymes involved in signal transduction, including protein kinase A by anchoring to AKAP3 [3,4] or AKAP4 [5,6], the Rho signaling pathway through ropporin [7] and rhophilin [8]. It has also been implicated in calcium signaling because it contains CABYR [9,10], a polymorphic, testis-specific calcium binding protein that is tyrosine [9] as well as serine/threonine phosphorylated [11] during in vitro sperm capacitation. At least nine glycolytic enzymes, including glyceraldehyde 3-phosphate dehydrogenase (GAPDH), glyceraldehyde 3-phosphate dehydrogenase-2 (GAPDH-2) [12,13], hexokinase 1 (HK1) [14,15], isoform of aldolase 1 (ALDOA), lactate dehydrogenase A (LDHA) [16], triose phosphate isomerase (TPI), pyruvate kinase, lactate dehydrogenase-C (LDH-C), and sorbitol dehydrogenase (SDH) [16], have been localized to the human and/or mouse FS. Moreover, a unique ADP/ATP carrier protein, SFEC [AAC4] is co-localized with several glycolytic enzymes in the FS [17]. The presence of four ion channel proteins, CatSper 1-4, in the membrane of the principal piece [18] overlying the FS in which CABYR is found, has led to hypotheses that CABYR plays a role in calcium signaling. Thus, observations indicate that the FS plays important roles in energy metabolism, ATP generation for sperm motility, calcium signaling, and as a scaffold for signaling molecules in addition to its role as a structural girdle surrounding the outer dense fibers and axoneme. A model has been proposed in which the FS represents a highly ordered complex, somewhat analogous to the electron transport chain, in which adjacent enzymes in the glycolytic pathway are assembled to permit efficient flux of energy substrates and products, possibly as a nucleotide shuttle between flagellar glycolysis, protein phosphorylation and mechanisms of motility [17].

Both mouse and human CABYRs are polymorphic proteins. Four murine CABYR variants, orthologous to human CABYR forms I, III, IV and VI, have been identified, which consist of two coding regions, CR-A and CR-B. The murine pattern is similar to that of human CABYR cDNAs of which six isoforms, three involving CR-B, are known. In the mouse, two stop codons (TGA and TGA) followed by six in-frame nucleotides separate CR-A from CR-B resulting in 453 and 199 aa reading frames, respectively [19].

While both the genomic structure and RNA splicing of murine CABYR have been reported, information on CABYR dynamic expression in mouse spermatogenesis is lacking. Moreover, because of the complexity of this polymorphic protein, there are no data on how CABYR isoforms associate and assemble into the FS.

It has been identifed that CABYR, ROPN1, ASP and SP17 share high sequence conservation with the PKA regulatory subunit's (RII) dimerization/docking (R2D2) domain, which binds the amphipathic helix region of AKAPs [20,21]. Moreover, CABYR has been shown to co-precipitate from sperm lysate with AKAP3 [22,23], and recently, evidence has been provided that the aforementioned proteins interact with a variety of AKAPs and that the interaction of CABYR with AKAPs appears to be much more limited [24]. However, further research on the relationships between CABYR and other FS proteins besides of AKAPs will facilitate the understanding of the basic physiology of FS. Interestingly, in our previous study, a novel protein named FSCB was found to co-imunoprecipitate with CABYR in sperm lysates [25].

In the present study, the capacity of the multiple CABYR isoforms to associate into dimers and oligomers, and the relationships between CABYR and other FS proteins such as the AKAPs and ropporin were studied in the mouse. Full-length murine CR-A and CR-B were cloned and expressed as recombinant proteins CABYR-A and CABYR-B. Antibodies to each protein isoform were produced and employed in 2-D diagonal gel electrophoresis and immunoblotting to determine which CABYR isoforms are involved in CABYR assemblies. Co-immunoprecipitations with polyclonal or monoclonal antibodies to CABYR variants, AKAP3 and AKAP4 were performed and 2-D gel immunoblots were analyzed to identify CABYR partner proteins, interactions of which were studied further by yeast two-hybrid analyses.

## Methods

### Cloning and sequencing of mouse CR-A and CR-B cDNA

Mouse CABYR gene-specific primers were designed to create an Nco I site at the 5' end and a Not I site at the 3' end of PCR amplicons encompassing the full length coding region A (CR-A) (nucleotide numbers 73-1431 encoding 453 amino acids) or coding region B (CR-B) (nucleotide numbers 1449-2039 encoding 193 amino acids) using the mouse CABYR form I (GenBank accession number AF359382) and form III (accession number AF359383 cDNA sequences). Primers for mouse CR-A (forward primer: 5'-CAT GCC ATG GTT TCT TCA AAG CCC AGA CTT-3'; reverse primer: 5'-ATA GTT TAG CGG CCG CAA CCT GTT CAG GAG CAG CTT CCC C-3') and CR-B (forward primer: 5'-CAT GCC ATG GCA ACA AGC GAA GCA GGA CAA CCA-3'; reverse primer: 5'-ATA GTT TAG CGG CCG CAG GTT CTG CTC TGC GGA CAT GGG C-3') were obtained from GIBCO BRL (Life Technologies, Carlsbad, CA). PCR was performed with 0.2 ng of mouse testicular Marathon-ready cDNA (BD Biosciences-Clontech, San Jose, CA) in a 50 μl assay system in a MJ Research (Waltham, MA) thermal cycler (PTC-200 DNA engine) using a program of one 6 min cycle at 94°C followed by 35 cycles of denaturation, annealing and elongation at 94°C for 1 min, 60°C for 1 min and 68°C for 3 min, respectively. The 1359 bp CR-A and 591 bp CR-B amplicons were separated on a 1% NuSieve (FMC Bio-Products, Rockland, ME) agarose gel, subcloned into the pCR 2.1-TOPO vector (Invitrogen, San Diego, CA), and sequenced in both directions to confirm authenticity, using vector-derived and insert-specific primers.

### Expression and purification of murine CABYR-A and CABYR-B recombinant proteins and antibody production

Mouse CR-A and CR-B were cloned into the pET28b+ expression vector and transformed into E. coli BLR (DE3) host strain (Invitrogen, San Diego, CA), and recombinant CABYR-A and CABYR-B, each with six His residues at the C-terminus, were expressed. Female guinea pigs were immunized with purified recombinant CABYR-A or CABYR-B protein (100 μg/animal) in Freund's complete adjuvant. Animals were boosted three times at intervals of 14 days with 50 μg of recombinant protein in incomplete Freund's adjuvant. Guinea pigs were sacrificed after confirming the presence of serum antibody by Western blot analysis using recombinant CABYR-A, CABYR-B and mouse sperm proteins. These studies were conducted under protocols 1574 and 2545 approved by the Institutional Animal Care and Use Committee at the University of Virginia in accord with the humane use of animals in research.

In our preliminary studies, it was found that the rat anti-human recombinant AKAP3 polyclonal antibody which was previously reported [4] can recognize mouse AKAP3 protein (data not shown). Therefore, the rat anti-human recombinant AKAP3 polyclonal antibody was used in the present study of mouse proteins. Mouse anti-mouse monoclonal AKAP4 antibody was purchased from BD Biosciences Pharmingen (San Diego, CA).

### Non-reducing and reducing 1-D SDS-PAGE of mouse sperm proteins

Mouse cauda epididymal sperm were collected from 12-15 week old mice (ICR strain, Harlan Sprague-Dawley, San Diego, CA). 5 × 10^8 ^sperm/ml were routinely solubilized in Celis lysis buffer [26] consisting of 9.8 M urea, 2% NP-40, and a complete protease inhibitor cocktail (Roche), with (reducing condition) or without (non-reducing condition) 100 mM DTT or 5% β-mercaptoethanol (β-ME), by constant shaking at 4°C for 30-60 min. Samples were heated at 100°C for 5 min, insoluble material was removed by centrifugation at 16,000 ×g for 5 min, and the supernatants were applied to 1-D SDS-PAGE.

### 2-D IEF-SDS/PAGE of mouse sperm proteins

200 μl sperm extract by Celis lysis buffer with 100 mM DTT (1 × 10^8 ^sperm/ml) was applied to the first dimension after addition of 2% v/v Ampholines (pH 3.5-10). Isoelectric focusing (IEF) was performed using a Protean IEF cell and an 11 cm tray (Bio-Rad, Hercules, CA, USA). Nonlinear strips (11 cm, pH 3-10) were rehydrated at 50 V for 12 h and then linearly increased to 8,000 V for a total of 30,000 V·h. For second dimension SDS-PAGE, the IPG strips were first incubated in equilibration buffer containing 37.5 mM Tris-HCl (pH 8.8), 6 M urea, 4% w/v SDS, 20% glycerol, and 100 mM DTT for 20 min. The equilibrated IPG strips were then transferred onto Criterion 4-15% linear gradient gels (Bio-Rad, Hercules, CA, USA) and electrophoresis was carried out at room temperature using a Criterion gel system (Bio-Rad, Hercules, CA, USA). Immunoprecipitates with various antibodies were separated by 2-D IEF-SDS/PAGE and identified by Western blot and mass spectrometry.

### 2-D nonreducing/reducing diagonal electrophoresis

Diagonal SDS-PAGE was performed as described by Sommer, et al [27] employing SDS-PAGE gradient gels (4-15%) in large format (20 × 16 cm, Protean II XL, Bio-Rad) and standard electrophoretic settings, using 1.0 mm or 1.5 mm thick gels for the first and second dimensions, respectively. In all assays, 100-200 μg samples of Celis-extracted sperm lysates (without β-ME and DTT) were diluted 1:1 with 2× sample buffer (without β-ME and DTT) and heated at 100°C for 5 min immediately preceding sample application to the first dimension. After the initial non-reducing electrophoresis, the entire lane containing the resolved proteins was excised, placed into containers with reducing buffer (containing 100 mM DTT or 5% β-ME), and incubated at 22°C for 15 min. The strips were then placed horizontally on top of the second dimension gradient gels (4-15%), overlaid with the SDS buffer containing β-ME to provide reducing conditions, and electrophoresis was carried out at room temperature. Proteins in the gels were either stained with Coomassie Brilliant Blue R-250 or silver nitrate for image analysis or were transferred to PVDF membranes for Western blotting.

### Immunoblotting

Proteins were transferred from unstained gels to PVDF membranes using a Bio-Rad Trans Blot Electrophoretic Transfer Cell (Bio-Rad Laboratories, Hercules, CA) according to the manufacturer's instructions. Membranes were blocked with 5% nonfat milk in PBS for 1 h at room temperature, washed three times with PBS-Tween (0.05% Tween-20 in PBS) and incubated overnight at 4°C with 15 ml of a previously determined working dilution of guinea pig pre-immune or immune serum (anti-CRA serum at 1:3000, anti-CRB serum at 1:1000). After washing thrice, the membranes were incubated with HRP-conjugated goat anti-guinea pig immunoglobulin (Sigma-Aldrich Quimica S. A. Madrid, Spain), washed × 3, and immunoreactive spots were detected by ECL (Amersham Pharmacia Biotech, Sunnyvale, CA) as described [8] or were visualized with the colorimetric reagent 3, 3', 5, 5'-tetramethylbenzidine (TMB) (Kirkegaard and Perry, Gaithersburg, MD).

### Indirect immunofluorescence localization of CABYR-A in the seminiferous tubules and epididymis

Testes and epididymides were obtained from 12-15 week-old mice (ICR strain, Harlan Sprague-Dawley, San Diego, CA) and fixed in Bouin's solution overnight at room temperature. They were embedded in paraffin, serially sectioned at 5 μm. For staging of tubule sections, periodic acid-Schiff (PAS) staining was performed following the standard protocol.

For immunostaining, after de-paraffination and rehydration, adjacent sections were incubated in 2 N HCl for 20 min, washed 3 × 5 min in PBS with 0.05% Tween 20 (PBS-T), and incubated in blocking solution containing 10% normal goat serum (NGS) in PBS-T for 30 min. The preparations were then incubated with anti-CABYR-A antiserum or pre-immune serum (1:400) in PBS-T containing 1% BSA (PBS-T-BSA) for 1 h at room temperature or overnight at 4°C, washed 3 × 5 min in PBS-T, incubated with Cy3-labeled goat anti-guinea pig IgG (1:400; Jackson Immuno-Research, West Grove, PA) in PBS-T-BSA, washed 3 × 5 min in PBS-T, and mounted with Slow Fade (Molecular Probes, Eugene, OR). PAS stained sections were observed by light microscopy and each tubule cross section was scored for the stage of the cycle of the seminiferous epithelium according to the criteria of Russell, et al [28]. Adjacent serial sections were observed by fluorescence microscopy to determine the corresponding immuno-staining pattern of the seminiferous epithelium. Red fluorescent images were recorded with a Zeiss digital camera and compiled using Openlab software (Improvision, Inc., Boston, MA). The immuno-staining to the caudal epididymal sperm is also conducted by the same method.

### Localization of CABYR-A in mouse sperm by immunoelectron microscopy

Caudal sperm from adult Swiss mice were retrieved and immunostained following the reference [25]. Guinea pig antiserum against CABYR-A and the pre-immune serum was diluted by 1:100. 5 nm gold-conjugated secondary antibodies, goat anti-guinea pig IgG (Goldmark Biologicals, Phillipsburg, NJ) was diluted by 1:50. The stained sections were observed with a 100CX electron microscope (JEOL, Peabody, MA).

### Co-immunoprecipitation of mouse sperm using anti-CABYR-A, anti-CABYR-B, anti-AKAP3 polyclonal antibody or anti-AKAP4 monoclonal antibody

To optimize immunoprecipitations of CABYR and partner proteins from mouse sperm extracts, two methods were used. Method 1: 2 × 10^8 ^epididymal sperm were suspended in 1 ml lysis buffer (10 mM Na_2_HPO_4_, 50 mM β-glycerophophate, 50 mM NaF, 1 mM EDTA, 1 mM EGTA, 1 mM DTT, 10 μg/ml leupeptin, 10 μg/ml pepstatin, 10 μg/ml aprotinin, 1 mM PMSF, 0.1% Tween 20) at 2-8°C, and the protocol described in the Immunoprecipitation Kit (Roche Applied Science, Indianapolis, USA) was followed. Immunoprecipitates on agarose pellets from one set of experiments, including the anti-CABYR-A and pre-immune control, were microsequenced by mass spectrometry. Another set of immunoprecipitates was retrieved by elution from protein A-agarose using 200 μl of Celis buffer followed by denaturation at 100°C for 3 min, 2-D gel electrophoresis, and silver staining or Western blotting. Method 2: Since AKAP3 and AKAP4 proteins were not thoroughly extracted using the lysis buffer described in method 1 above in initial studies, a modified immunoprecipitation strategy to extract low solubility proteins was followed. 8 × 10^8 ^sperm were resuspended in 2 ml Celis buffer with Complete Protease inhibitor cocktail without DTT, and the suspension was incubated for 0.5-1 h at 4°C on a rocking platform. Following centrifugation at 4°C, 12,000 ×g, in a tabletop microfuge for 10 min to remove debris, the supernatant was transferred into a dialysis cassette with 10 kDa cut off (Pierce) and dialyzed against 1 × PBS solution for 24h at 4°C with two changes of PBS. The dialyzed suspension was centrifuged at 4°C, 6,000 ×g for 10 min to remove the precipitates. The suspension was then transferred to four 1.5 ml tubes and the manufacturer's immunoprecipitation protocol followed as in method 1. Immunoprecipitates were retrieved by elution from the protein A-agarose pellet with 200 μl of Celis buffer or with 50 μl of 2 × Laemmli sample buffer, and proteins were denatured at 100°C for 3 min. The protein A-agarose was centrifuged at 12,000 ×g for 20 s at 15-25°C in a microfuge, and supernatants were transferred to a fresh tube for 2-D gel IEF-SDS/PAGE. Western blots were performed as described above.

### Tandem mass spectrometry peptide sequencing

2-D gel spots from immunoprecipitates with immune sera were compared to those with pre-immune sera. Spots appearing in the experimental samples and not in the pre-immune samples were cut out of the 2-D gel using fine coring tools [29], in-gel digested by trypsin overnight at 37°C, and microsequenced by tandem mass spectrometry at the W. M. Keck Biomedical Mass Spectrometry Laboratory of the University of Virginia. The data were analyzed by database searching using the Sequest search algorithm against the Non-redundant database.

### Yeast two-hybrid assay

Vectors, yeast, and major reagents were supplied as part of the Matchmaker Gal4 Two-Hybrid System 3 (Clontech, Palo Alto, CA). Gene segments were obtained by PCR using 0.2 ng of mouse testicular marathon-ready cDNA (BD Biosciences-Clontech) as the template and the primers listed in Table 1. The full-length open reading frame of CR-A (79-1431), a deletion construct of a truncated CR-A (TCR-A) without the RII-like domain (223-1431), and the full-length open reading frame of CR-B (1443-2039) were cloned into the pGADT7, while full-length open reading frames of AKAP3 (292-2883), AKAP4 (148-2664) and ropporin (1-636) were cloned into the pGBKT7 vectors using standard cloning procedures. Yeast strain AH109 was simultaneously co-transformed with two recombinant plasmids having different selection markers using the LiAc-mediated yeast transformation as described in the Yeast Protocols Handbook (PT3024-1; Clontech). In this two-hybrid system, the GAL4 BD (binding domain) binds to the GAL upstream activating sequence and, if the fusion proteins interact, the AD (activating domain) is brought into proximity with the promoters of four reporter genes (HIS3, ADE2, MEL1, and lacZ), thereby activating transcription and permitting growth on selection media (His- and Ade-) and the expression of α-galactosidase (MEL1 product) and β-galactosidase (lacZ product). Co-transformed yeast cells were isolated by growth on SD/-Leu/-Try plates at 30°C for 3 days. For medium stringency or high stringency selection, cells were then transferred to SD/-His/-Leu/-Trp or SD/-Ade/-His/-Leu/-Trp plates, supplemented with 20 μg/ml X-α-Gal (5-bromo-4-chloro-3-indolyl-α-D-galactopyranoside), and allowed to grow at 30°C for 3-5 days to select for colonies that expressed interacting proteins.

**Table 1:** Construction of Gal4 fusions of mouse genes for yeast two-hybrid assay

Construction	Nucleotide accesion numbers in GenBank	Nucleotides	Primers used in PCR	Designed Restrictive Enzymes
pGADT7-mCRA	AF359382	73-1431	5'-CGGGATCCAGATGATTTCTTCAAAGCCC AGAC-3' 5'-CCGCTCGAG TCAAACCTGTTCAGGAGCAGCTT-3'	BamH I Xho I
pGADT7-mTCRA	AF359382	223-1431	5'-CGGGATCCACGGGAATTCGTCTCTAGATATAA-3' 5'-CCGCTCGAGTCAAACCTGTTCAGGAGCAGCTT-3'	BamH I Xho I
pGADT7-mCRB	AF359382	1443-2039	5'-CGGAATTCGCACTAGCAACAAGCGAAGCAGG-3' 5'-CCGCTCGAGTTAAGGTTCTGCTCTGCGGACA-3'	EcoR I Xho I
pGBKT7-mAKAP3	NM_009650	292-2883	5'-CGGAATTCATGGCGGATAGGGTTGACTG-3' 5'-ACGTCGACCAGGTTTGCCATCAGCCAGTCC-3'	EcoR I Sal I
pGBKT7-mAKAP4	NM_009651	148-2664	5'-CGGAATTCATG TCTGATGACA TTGACTGGT-3' 5'-ACGTCGACCAGGTTAGCGAGAAGCCAGTCC-3'	EcoR I Sal I
pGBKT7-ropporin	AF178531	1-636	5'-CATGCCATGGGACCTCAGACAGACAAGCAAG -3' 5'-CGGAATTCTTATTCCAGCCGAACCCTAGGGT-3'	Nco I EcoR I

### α-Galactosidase quantitative assay

SD/-Leu/-Trp or SD/-His/-Leu/-Trp cultures were inoculated with a single, fresh yeast colony and incubated overnight at 30°C. The A_600 _was measured, and the supernatant was harvested by centrifugation at 14,000 ×g for 2 min. 40 μl of the supernatant was combined with 120 μl of fresh assay buffer [2:1 ratio of 0.5 M sodium acetate, pH 4.5, to 100 mM p-nitrophenyl-D-galactopyranoside (Sigma) ] and incubated at 30°C for 60 min. The reaction was stopped by addition of 840 μl of 0.1 M Na_2_CO_3_. Optical density was measured at 410 nm in a 1.5-ml cuvette, and α-galactosidase units were calculated from the formula: [milliunits/(ml × cell)] = A_410 _× 1000 μl × 1000/[16.9 (ml/μmol) × 60 min × 40 μl × A_600_] (Yeast Protocols Handbook). Each yeast colony was assayed in triplicate.

## Results

### Generation of recombinant CABYR-A and CABYR-B proteins and anti-CABYR-A, anti-CABYR-B polyclonal antibodies

Mouse CABYR has three alternative splice variants involving two coding regions, CR-A and CR-B, which are separated by dual stop codons [19]. Affinity-purified recombinant full length CABYR-A and CABYR-B were used to generate polyclonal antisera (see Additional file 1, Fig. S1). The specificities of anti-CABYR-A and anti-CABYR-B antibodies were confirmed by Western blotting, which detected bands at about 80 kDa and 22 kDa, respectively. The recombinant murine CABYR-A protein, just like the native form of mouse CABYR-A [19], resembled recombinant human CABYR-A protein in migrating at a much higher molecular weight (MW) than that calculated theoretically [9]. Pre-immune serum for CABYR-A or CABYR-B did not immunoreact with either recombinant protein. Importantly, anti-CABYR-A antibody did not show any cross-reactivity with recombinant CABYR-B nor did anti-CABYR-B cross react with CABYR-A protein. Thus, antibodies to CABYR-A and CABYR-B demonstrated specificity for their respective isoforms (see Additional file 2, Fig. S2).

### Polymorphism and microheterogeneity of mouse CABYR proteins

**Figure 1:**
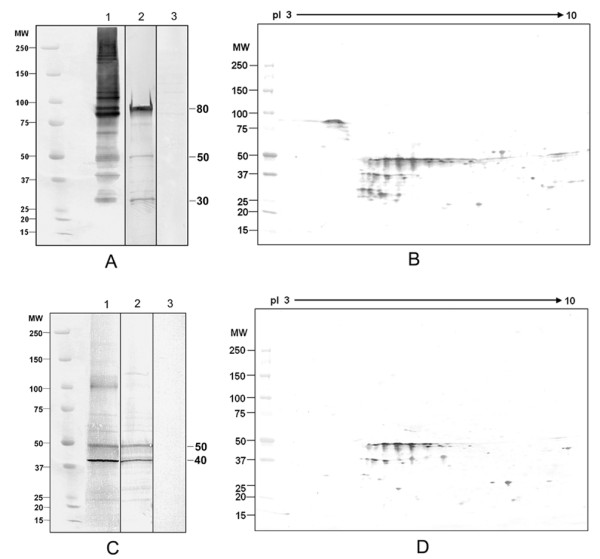
**Western blots analysis of CABYR oligomers in mouse sperm**. Immunoreactive CABYR isoforms were observed after non-reducing and reducing 1-D PAGE or 2-D PAGE using antisera specific to mouse CABYR-A and CABYR-B. CABYR-A containing isoforms (A) migrated in the non-reduced state (lane 1) as both high and low molecular weight complexes, with bands particularly prominent at about 200, 100, and 80 kDa. Upon reduction (lane 2), the immunoreactive CABYR-A containing isoforms migrated at 80, 50, and 30 kDa. CABYR-B (C) containing isoforms in the non-reduced state (lane 1) showed immunoreactive bands at 200, 100, 50, and 40 kDa, and upon reduction (lane 2) the major immunoreactive bands ran at 50 and 40 kDa with a high molecular weight complex at 100 kDa being noticeably absent upon reduction. In both cases, strips incubated with the corresponding pre-immune control sera were negative (A3, C3). 2-D Western blots of mouse CABYR-A (B) and CABYR-B (D) showed that the pI 4.0, 80 kDa spot is the dominant CABYR-A only variant. This isoform displays a modest degree of charge and mass polymorphism compared to the other variants located in the neutral region, which contain both CABYR-A and CABYR-B and show a remarkable degree of charge and mass polymorphism. All protein spots that were immunoreactive for CABYR-B (D) aligned with CABYR-A immunoreactive spots (B). The absence of spots unique to CABYR-B stained gels indicates that proteins containing only CABYR-B are not translated in mouse sperm.

Six alternative splice variants of CABYR have been reported in humans, but only four murine CABYR variants have been identified, two of which splice into CABYR-B [19]. To test the hypothesis that some of these CABYR variants form oligomers which might participate in the assembly of the supra-molecular structure of the FS, non-reduced and reduced sperm protein extracts were separated by 1-D SDS-PAGE and immunoblotted with sera to CABYR-A and CABYR-B, respectively. Non-reducing lanes (Fig. 1A, lane 1) contained numerous high molecular weight immunoreactive CABYR-A containing bands, which upon reduction migrated at lower molecular weights, mainly about 80 kDa, 50 kDa and 30 kDa (lane 2), indicating that CABYR-A containing variants formed oligomers. Non-reduced sperm proteins stained with anti-CABYR-B showed a prominent immunoreactive band above 100 kDa as well as 50 and 40 kDa bands. This 100 kDa band disappeared upon reduction while the 50 and 40 kDa bands persisted. This indicated that CABYR-B containing variants also formed oligomers. Some CABYR bands of identical molecular weight were recognized by both anti-CABYR-A and anti-CABYR-B sera under non-reducing conditions. For example, the mouse CABYR band of ~100 kDa was identified under non-reducing conditions by both anti-CABYR-A (Fig.1A, lane 1) and anti-CABYR-B antibodies (Fig. 1C, lane 1), being more strongly recognized by the anti-CABYR-A antibody. Upon reduction, this ~100 kDa band disappeared, while the relative abundance of the 50 kDa band which was recognized by both anti-CABYR-A and anti-CABYR-B sera increased significantly. Comparison of 1-D and 2-D Western blot results suggested that the 50 kDa band or spots represent a component monomer of the 100 kDa band, and it is reasonable to speculate the 100 kDa band may represents a homodimer of the CABYR-A and CABYR-B containing 50 kDa variant. Based on immunoreactivity on 1-D gels, the 80 kDa monomer containing only CABYR-A was the most abundant CABYR isoform in sperm, followed by the CABYR-A and CABYR-B containing 50 kDa variant.

The 2-D Western blots of mouse sperm with anti-CABYR-A (Fig. 1B) and anti-CABYR-B (Fig. 1D) showed that CABYR isoforms containing both CABYR-A and CABYR-B comprised a remarkable number of charge and mass variants. This protein microheterogeneity was particularly prominent in the pI range 5 to 8, while the pI 4, 80 kDa CABYR-A-only variant displayed fewer charge variants. All spots that were immunoreactive with CABYR-B antisera also reacted with anti-CABYR-A, suggesting that CABYR mRNA that contains only CR-B is not translated in mouse sperm (Fig. 1B, D).

### Relationships between CABYR monomers and oligomers

**Figure 2:**
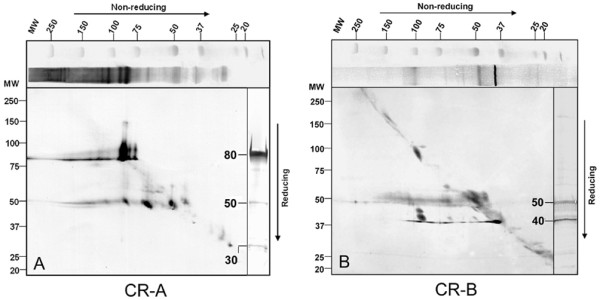
**2-D diagonal gel Western blot of mouse sperm extract**. A: Immunoblots probed with guinea pig anti-CABYR-A polyclonal antibodies. The presence of spots below the diagonal showed that the band running at ~70 kDa under non-reducing condition was broken down to ~50 kDa under reducing conditions; ~110-130 kDa bands resolved into 80 kDa and ~50 kDa forms; and a ~200 kDa band decomposed into 80 kDa and 50 kDa components. B: Probing by guinea pig anti-CABYR-B polyclonal antibodies showed that most of the high MW bands under non-reducing condition were broken down to 50 kDa and 40 kDa under reducing conditions. It may be concluded that the 50 kDa band contains both CABYR-A and CABYR-B. Principal CABYR monomers migrate at 80, 50 and 40 kDa.

Mouse sperm Celis extracts were electrophoresed on 2-D diagonal gels [non-reducing followed by reducing dimensions] to study CABYR-A and CABYR-B containing monomers involved in CABYR oligomerization. The most intensely immunoreactive band on 1-D non-reducing blots stained with anti-CABYR-B sera (Fig. 2B) was a ~40 kDa protein. This 40 kDa protein was also present, albeit less immunoreactive, on non-reducing 1-D blots stained with CABYR-A antisera (Fig. 2A). When the reducing dimension was exposed to anti-CABYR-B (Fig. 2B), the 40 kDa protein migrated on the diagonal, indicating it is a CABYR monomer. Blots also revealed a ~50 kDa band under non-reducing conditions that immunoreacted with both CABYR-A and CABYR-B (Fig. 2A &2B), although more strongly with anti-CABYR-B. This 50 kDa band ran at ~50 kDa under reducing conditions on both diagonal blots, confirming that the 50 kDa band also represents a CABYR monomer. Similarly, the ~80 kDa band (Fig. 2A), that under non-reducing conditions only reacted with anti-CABYR-A, continued to migrate under reducing conditions on the diagonal at ~80 kDa, indicating the presence of an 80 kDa CABYR monomer containing only CABYR-A. Thus in mouse sperm CABYR protein monomers that migrate at 80, 50 and 40 kDa appear to predominate.

Several immunoreactive CABYR bands are noted in non-reducing gels that decompose with reduction into subunits (Fig. 2). For example, a ~70 kDa band, noted on both blots under non-reducing conditions, resolved under reducing conditions into a 50 kDa band that reacted with both anti-CABYR-A and anti-CABYR-B, suggesting that the 70 kDa form was composed of one 50 kDa variant and another CABYR variant (or one of its binding partners) of ~20 kDa. Similarly, a ~100 kDa immunoreactive band resolved into 50 kDa monomers, suggesting the 100 kDa oligomer is very likely a homodimer of the 50 kDa CABYR-A and CABYR-B containing variant, which is consistent with the data shown in Fig 1. In addition, many high molecular weight bands ranging from ~110-250 kDa resolved into 80 kDa and 50 kDa bands upon reduction, indicating that a set of high molecular weight CABYR heterodimers are present in mouse sperm and that they are composed of one or more 80 kDa variants (containing CRA only) and one or more 50 kDa variants (containing both CRA and CRB). Thus, CABYR forms heterodimers mainly from the 80 kDa (CABYR-A only) and 50 kDa variants (containing both CABYR-A and CABYR-B) which then form larger assemblies that participate in the supra-molecular structure of FS.

**Figure 3:**
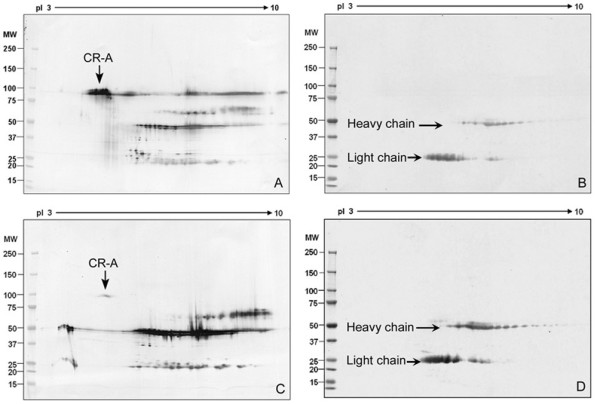
**CABYR-A only variant can be co-immunoprecipitated with CABYR-B containing variants**. A, B: 2-D Western blots of proteins immunoprecipitated using anti-CABYR-A polyclonal antibody (A) or pre-immune serum (B) and probed by anti-CABYR-A primary antibody and anti-guinea pig secondary antibody. Heavy and light chain reactive spots are noted in pre-immune controls (B & D). IP with anti-CABYR-A brought down soluble forms of CABYR mainly including 80 and 50 kDa isoforms. The location of the 80 kDa CABYR-A only isoform is indicated by an arrow (A). The 80 and 50 kDa isoforms of mouse CABYR appear to be the most soluble and readily extracted. C, D: 2-D Western blots of mouse sperm immunoprecipitate using anti-CABYR-B polyclonal antibody (C) or pre-immune serum (D) probed by anti-CABYR-A polyclonal antibody. The 80 kDa spot indicated by an arrow (C) is a CABYR-A only variant and is co-immunoprecipitated with CABYR-B, demonstrating that the 80 kDa CABYR-A only variant complexes with CABYR-B containing variants.

Immunoprecipitations using the standard buffer (Roche Applied Science, Indianapolis, USA) were conducted to determine which isoforms of CABYR are soluble and whether complexes containing CABYR-A isoforms could be precipitated by anti-CABYR-A or B antibodies. Following immunoprecipitation with anti-CABYR-A, the CABYR-A containing isoforms recovered from protein A were studied on Western blots reacted with preimmune sera (Fig. 3B) or with anti-CABYR-A (Fig. 3A). A range of CABYR isoforms were noted in the immunoprecipitates on the 2-D-Western blots in Fig. 1B, revealing that CABYR complexes are soluble under these relatively mild extraction conditions in the lysis buffer. The predominant protein precipitated by the anti-CABYR-A antibody was the 80 kDa CABYR-A only variant, confirming the solubility of this isoform. When the anti-CABYR-B antibody was used for immunoprecipitation to explore the potential complexes of CABYR-B containing isoforms with CABYR-A containing isoforms, the repertoire of CABYR-A containing proteins that was recovered included groups at 40 to 50 kDa and, most importantly, the 80 kDa CABYR-A only variant was co-immunoprecipitated along with the CABYR-B containing isoforms. These results further confirm the presence of soluble CABYR complexes that contain both CABYR-A only isoforms and CABYR-A and CABYR-B isoforms.

### Localization and dynamic expression patterns of CABYR-A in the seminiferous epithelium

**Figure 4:**
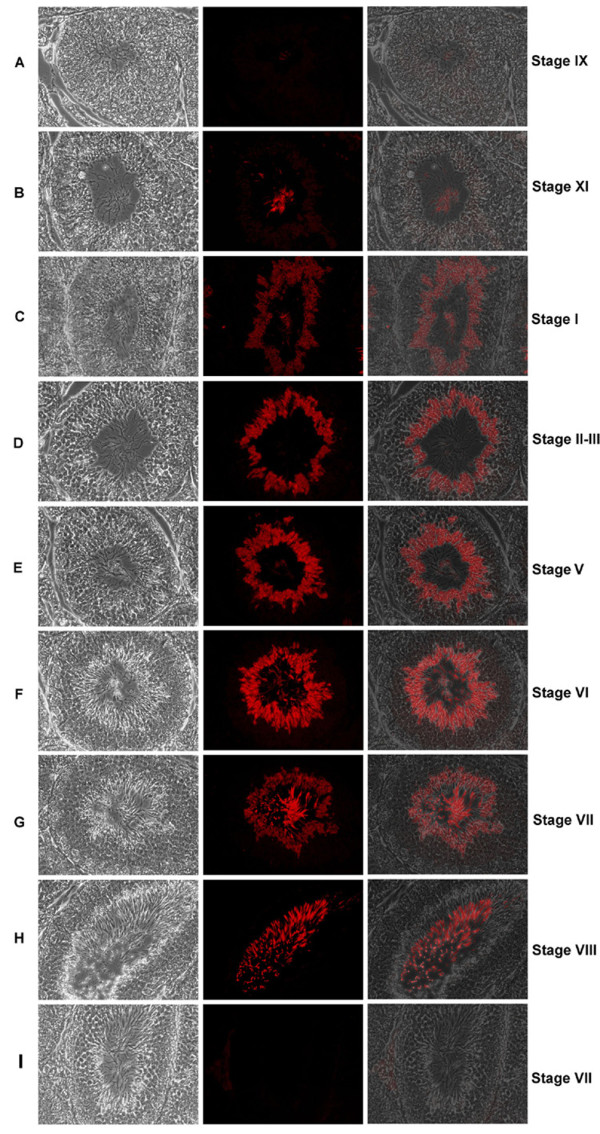
**The dynamic expression of CABYR-A in mouse spermiogenesis**. Immunofluorescent localization of mouse CABYR-A in paraffin sections of mouse testis were studied by using anti-CABYR-A polyclonal antibody (A-H) or pre-immune control (I), and Cy3-conjugated secondary antibodies. Column 1: Bright field phase images; Column 2: Corresponding indirect immunofluorescence; Column 3: Overlay of 1 and 2. No red fluorescence is observed in the spermatids in steps 9 and 10 (stage IX-X of the cycle) (A). Very faint red fluorescence representing CABYR-A protein is first visible in the cytoplasm of step 11-12 elongating spermatids (stage XI-XII) (B). CABYR protein becomes progressively more intense in the spermatid cytoplasm from step 13 (stage I) through step 15 (stage VI) (C-F). Staining is first noted in the sperm flagellum at step 15 (stage V-VI) (F) and appears in the principal piece of the flagellum at early step 16 (stage VII) accompanied by decreasing staining in the spermatid cytoplasm (G). Cytoplasmic staining of round spermatids was not evident and red fluorescence was found only in the principal piece of spermatid flagellum at late step 16 (stage VIII) (H). No immunofluorescence was observed with the pre-immune antiserum at any stage of spermatogenesis (I).

CABYR-A containing proteins were localized at each stage of the cycle of the seminiferous epithelium in tubular cross-sections by immunofluorescence microscopy. CABYR-A in mouse testes was restricted to post meiotic spermatids in the late steps of spermiogenesis, and the staining patterns showed dynamic and cyclic changes and migration during spermiogenesis. No fluorescent signals were detected in spermatogonia, spermatocytes, round spermatids or step 9-10 elongating spermatids (stage IX-X) (Fig. 4A). Red immunofluorescent signals for CABYR-A first appeared in the cytoplasm of elongating spermatids at step 11-12 as very faint staining in the seminiferous epithelium (stage XI-XII) (Note that the luminal sperm show a very strong red immunofluorescent signal in these preparations, Fig. 4B. Readers may wish to expand digital images to view the faint staining to best advantage.). Staining gradually became more dense and prominent in the spermatid cytoplasm from step 13 (stage I) through step 15 (stage VI) (Fig. 4C-F). At step 15 (stage V-VI) (Fig. 4F), strong immunofluorescence of spermatid flagella was noted. This flagellar staining increased from step 15 to step 16, becoming confined to the principal piece of late spermatids lining the surface of seminiferous epithelium (stage VII-VIII) (Fig. 4H). Concomitantly, cytoplasmic staining of spermatids decreased (Fig. 4G) and disappeared (Fig. 4H) from the seminiferous epithelium. At stages IX and X, when spermatids were released into the lumen, the seminiferous epithelium no longer showed any CABYR fluorescence and staining was observed only in the principal piece of luminal spermatids (Fig. 4A). Pre-immune serum from the same animal did not stain the seminiferous epithelium at any stage of the cycle (Fig. 4I).

### CABYR-A localizes to the FS of the principal piece in mature mouse sperm flagellum

**Figure 5:**
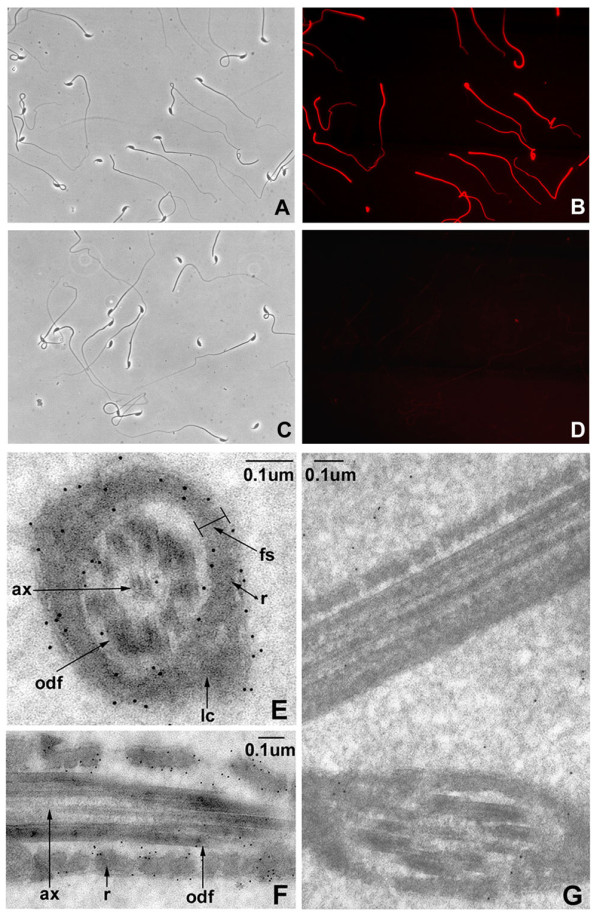
**Immunofluorescence and immunoelectron microscopic localization of mouse CABYR-A to the FS of principal piece on sperm flagellum**. A, C: Phase contrast images; B, D: Indirect immunofluorescence staining using anti-CABYR-A serum (B) or pre-immune serum (D). Intense fluorescent staining of mouse CABYR-A using the anti-CABYR-A antiserum is restricted to the principal piece of all mouse sperm flagellum in B (×400). Pre-immune serum did not stain the spermatozoa (D ×400). E, F: immunoelectron microscopic localization with guinea pig anti-CABYR-A antibody. Most of the gold particles were located over the fibrous sheath (fs), including the surface of the longitudinal columns (lc) and ribs (r). Occasional gold particles were present at the surface of the outer dense fibers (odf). CABYR-A was not detected in the axoneme (ax) or the matrix (interior) of the outer dense fibers. G. Pre-immune serum controls with sperm in longitudinal and oblique cross sections show very few gold particles. Bar: 0.1 μm.

Antibodies against CABYR-A recognized the entire length of the principal piece of methanol-fixed and non-fixed epididymal spermatozoa with an intense signal by indirect immunofluorescence microscopy. No immunofluorescence was noted in the head, connecting piece and middle piece in mouse sperm (Fig. 5B). The preimmune serum did not stain the sperm (Fig. 5D).

To characterize in detail the ultrastructural localization of CABYR-A, freshly prepared mouse sperm were examined by immunogold electron microscopy. Most of the gold particles, indicative of the presence of protein containing CABYR-A, were located in the FS, mainly over its surface, including the inner and outer surfaces of the longitudinal columns and ribs. Smaller numbers of gold particles were present on the surfaces of the outer dense fibers. CABYR-A was not detected in the axoneme, interior of the outer dense fibers or the mitochondrial sheath (Fig. 5E, F). Pre-immune serum controls both in longitudinal and oblique cross section showed only very few gold particles. Thus, the ultrastructural data clearly showed that CABYR-A is an integral FS component of mature mouse sperm and most likely is located on the surface of the FS.

### CABYR binds with AKAP3 and AKAP4 in mouse sperm

**Figure 6:**
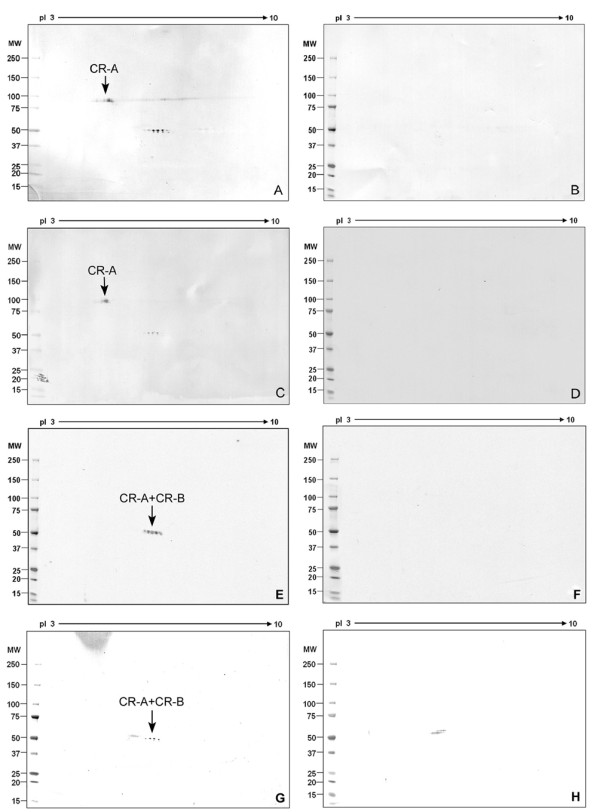
**Immunoprecipitation of CABYR protein isoforms by anti-AKAP3 and anti-AKAP4**. A, B: 2-D Western blots of immunoprecipitates from mouse sperm extracts using either anti-AKAP3 polyclonal antibody (A) or pre-immune serum (B) probed with anti-CABYR-A polyclonal antibody. CABYR-A protein isoform at 80 kDa, pI 4.5 is evident in immune complexes brought down by anti-AKAP3 but not by pre-immune serum. C, D: 2-D Western blot of immunoprecipitate from mouse protein extract using anti-AKAP4 monoclonal antibody (C) or normal mouse IgG (D) probed by anti-CABYR-A polyclonal antibody. The CABYR-A only variants of 80 kDa, pI 4.5 indicated by an arrow (C) were precipitated by anti-AKAP 4 antibody while normal mouse IgG immunoprecipitated no anti-CABYR immunoreactive proteins (D). 2-D Western blots of mouse sperm extract were immunoprecipitated using anti-AKAP3 polyclonal antibody (E), anti-AKAP4 monoclonal antibody (G), pre-immune serum (F), or normal mouse IgG (H) and probed by anti-CABYR-B polyclonal antibody. The 50 kDa, pI 6.5 spots indicated by arrows in E and G represent immunoprecipitated CABYR forms that contain both CABYR-A and CABYR-B and were recognized by antibody directed at CABYR-B.

To identify potential binding partners of CABYR, particularly scaffolding proteins of the FS, immunoprecipitation of sperm protein extracts was performed with anti-AKAP3 polyclonal antibody and pre-immune control serum (Fig. 6A, B) or anti-AKAP4 monoclonal antibody and normal mouse IgG control (Fig. 6C, D), followed by 2-D gel separation and Western blotting with anti-CABYR-A antibody. The 80 kDa CABYR-A only variant (arrow in Fig. 6A) as well as the 50 kDa isoforms containing both CRA and CRB (as shown in Fig. 1) were present in the mouse sperm extract immunoprecipitated using anti-AKAP3 polyclonal antibody but not when pre-immune serum was used (Fig. 6B). The 80 kDa and 50 kDa CABYR isoforms were also observed in immunoprecipitates obtained with anti-AKAP4 monoclonal antibody, but not when immunoprecipitation was conducted with normal mouse Ig G (Fig. 6C, D). These results indicate that the 80 kDa (CABYR-A only isoform) and 50 kDa isoforms (CABYR-A and CABYR-B containing) can be co-immunoprecipitated with both AKAP3 and AKAP4. Because there are more isoforms recognized overall with the anti-CABYR antisera than the few isoforms brought down in the anti-AKAPs immunoprecipitates, the 80 and 50 kDa isoforms are likely the main molecules that interact with the AKAPs.

When Western blots of immunoprecipitates obtained using anti-AKAP3 polyclonal antibody and anti-AKAP4 monoclonal antibody were performed with anti-CABYR-B, the 50 kDa isoforms were again noted, indicating that the 50 kDa isoforms contain both CABYR-A and CABYR-B and can be immunoprecipitated by anti-AKAP3 and anti-AKAP4 antibodies (Fig. 6E, G; controls in Fig. 6F, H). Thus both the 80 kDa CRA-only variants as well as the 50 kDa CABYR-A and CABYR-B containing variants participate in complexes containing AKAP3 and AKAP4. These results also indicate that relatively insoluble FS proteins like AKAP3 and AKAP4 were resolved and immunoprecipitated by the modified immunoprecipitation method used here.

### CABYR binding with ropporin

**Figure 7:**
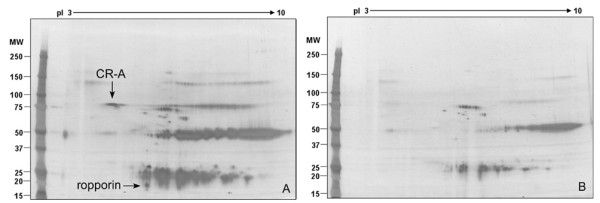
**Ropporin can be co-immunoprecipitated with CABYR**. 2-D silver stained gels after immunoprecipitating mouse sperm protein extracts with guinea pig anti-CABYR-A polyclonal antibody (A) or pre-immune serum (B) to identify possible CABYR binding partners. A silver stained spot present in A but absent in B was cored from gels and microsequenced by mass spectrometry, yielding peptides from mouse ropporin (accession number NP_109669).

CABYR can be partially extracted in non-ionic detergents such as NP-40, Triton-X 100, or Tween 20 although the majority of CABYR protein remains in the insoluble fraction (data not shown). Immunoprecipitation with anti-CABYR-A (Fig. 7A) or control pre-immune sera (Fig. 7B) was followed by 2-D analysis of the immunoprecipitates to detect CABYR binding partners. A unique spot at 20 kDa, pI 5 (Fig. 7A, arrow) was noted and cored from silver stained 2-D gels for microsequencing by mass spectrometry. The resultant peptide sequences, QVCIPPELPELLK, GEIPPVR, LIIHADELAQMWK, VLNLPTDLFNSVMNVGR, and FTEEIEWLK were found by database searching to belong to ropporin, or rhophilin associated protein 1 (NP_109669), which was therefore identified as a CABYR binding partner. These data support a participation of ropporin in CABYR complexes in mouse sperm.

### Confirmation of CABYR binding with AKAP3, AKAP4 and ropporin in mouse sperm by yeast two hybrid analysis

**Figure 8:**
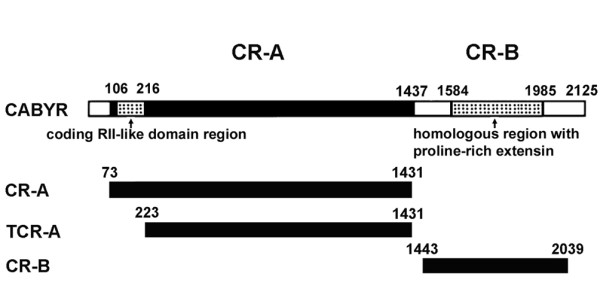
**A schematic diagram of mouse CABYR gene**. It illustrates the positions of different putative domains and the CABYR gene segments used for the construction of transformants (CR-A, TCR-A, and CR-B). The numbers refer to the nucleotide positions.

**Figure 9:**
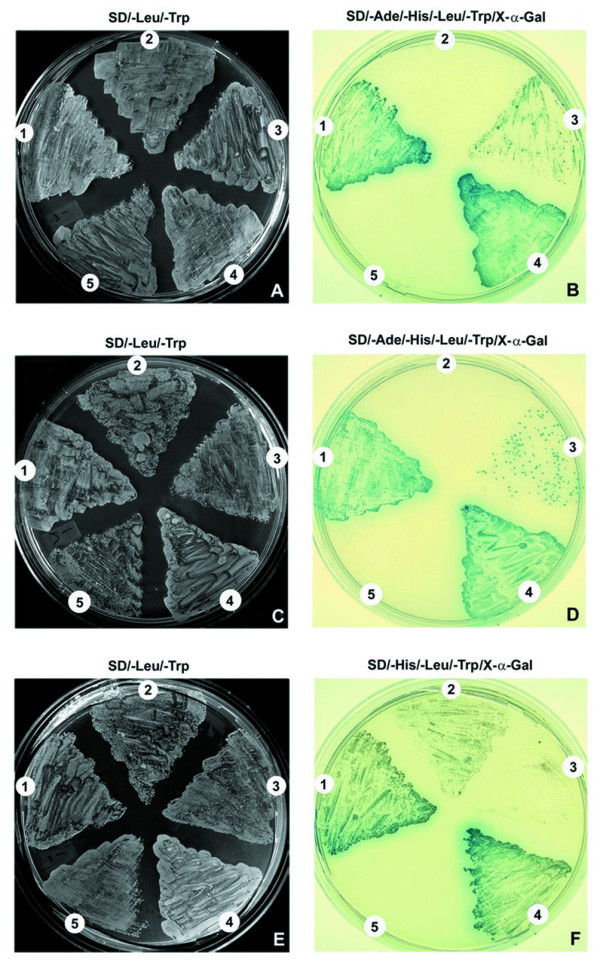
**Interactions between various CABYR constructs and AKAP3, AKAP4 or ropporin in the yeast two-hybrid system**. Yeast strain AH109 was co-transformed with pGADT7-CRA, pGADT7-TCRA or pGADT7-CRB and pGBKT7-AKAP3, pGBKT7-AKAP3 or pGBKT7-ropporin (pGADT7-T, while pGBKT7-53 served as a positive control and pGADT7-T and pGBKT7-Lam served as a negative control). The ability of the transformants to grow on selective SD/-Leu/-Trp plates (Fig. 11 A, C, E), SD/-Ade/-His/-Leu/-Trp plates (Fig. 11 B, D), or SD/-His/-Leu/-Trp (Fig. 11 F) was analyzed. A, B. Interaction between CABYR and AKAP3: 1, CR-A+AKAP3; 2, TCR-A+AKAP3; 3, CR-B +AKAP3; 4, p53 + Large T (positive control); 5, p53 + Lam C (negative control). While CABYR containing the RII domain showed signals equivalent to the positive control, the construct lacking the RII domain showed the signal equivalent to the negative control. C, D. Interaction between CABYR and AKAP4: 1, CR-A+AKAP4; 2, TCR-A+AKAP4; 3, CR-B +AKAP4; 4, p53 + Large T (positive control); 5, p53 + Lam C (negative control). The results are very similar to that of the interaction between CABYR and AKAP3. E, F. The analysis of interaction between CABYR and ropporin: 1, CR-A +ropporin; 2, TCR-A+ ropporin; 3, CR-B+ ropporin; 4, p53 + Large T (positive control); 5, p53 + Lam C (negative control). There is a weak interaction between CABYR-A, as well as truncated CABYR-A, and ropporin but not between CABYR-B and ropporin.

The RII like domain in CABYR is potentially critical for mediating binding with AKAP scaffolding proteins [9]. In mouse CABYR this domain is predicted to span amino acids 12 to 48 [19].To further test the interactions of mouse CABYR with AKAP3, AKAP4 and ropporin that are predicted by immunoprecipitation studies and to map regions that may possibly mediate the interactions of CABYR with other proteins, three different CABYR gene segments were tested in the yeast two hybrid system. Full length CR-A, a truncated CR-A lacking the putative RII domain, and full length CR-B were used for the construction of recombinant pGADT7 (Fig.8). Full-length open reading frames of AKAP3, AKAP4 and ropporin were used in recombinant pGBKT7 constructs. The abilities of the co-transformants to grow on selective SD/-Leu/-Trp plates (Fig. 9A, C, E), SD/-Ade/-His/-Leu/-Trp (Fig. 9B, D), or SD/-His/-Leu/-Trp (Fig. 9F) plates were analyzed. The results showed that the co-transformants with CR-A+AKAP3 (Fig. 9B-1) and CR-A+AKAP4 (Fig. 9D-1) grew in the selective SD/-Ade/-His/-Leu/-Trp medium to an extent comparable to the positive control (Fig. 9B, D-4). Importantly, when the X--Gal was applied to the top of the medium, both of these transformants gradually became blue as did the positive control after 3-5 days culture at 30°C. This result indicated that the reporter genes were activated by the interactions between full length CABYR-A and either AKAP3 or AKAP4.

Transformants with CR-B+AKAP3 (Fig. 9B-3) or CR-B+AKAP4 (Fig. 9D-3) also grew in the selective SD/-Ade/-His/-Leu/-Trp medium, but their growth was very slow and colonies were dispersed. These two co-transformants also turned blue with X-gal, but their color was much weaker than the positive control. Significantly, when the truncated CABYR construct that lacked the RII domain was used, the TCR-A +AKAP3 (Fig. 9B-2) or TCR-A+AKAP4 (Fig. 9D-2) transformants did not grow on selection medium. These results indicate that very strong interactions occur between mouse CABYR-A and both AKAP3 and AKAP4, and that this interaction is abolished by deletion of the CABYR-A RII domain. The results also suggest that a weak interaction occurs between CABYR-B and both AKAP3 and AKAP4, but this is much less significant than that mediated by the RII motif.

The combinations of CR-A+ropporin, TCR-A+ropporin, and CR-B+ropporin did not grow on the selective SD/-Ade/-His/-Leu/-Trp medium, but the first two did grow on the stringency SD/-His/-Leu/-Trp medium (Fig. 9F-1, 2) to an extent similar to the positive control (Fig. 9F-4). In contrast, the transformant with CR-B+ ropporin (Fig. 9F-3) did not grow on the SD/-His/-Leu/-Trp medium, thus resembling the negative control (Fig. 9F-5). These results suggest a weak interaction between CABYR-A, as well as truncated CABYR-A, and ropporin but not between CABYR-B and ropporin. Interestingly, although the interaction between CABYR-A and AKAP3/AKAP4 was abolished by deletion of the RII domain, deletion of this domain diminished but did not totally abolish the interaction between CABYR-A and ropporin, suggesting that CABYR-A/roporrin interactions may involve domains outside the RII motif.

**Figure 10:**
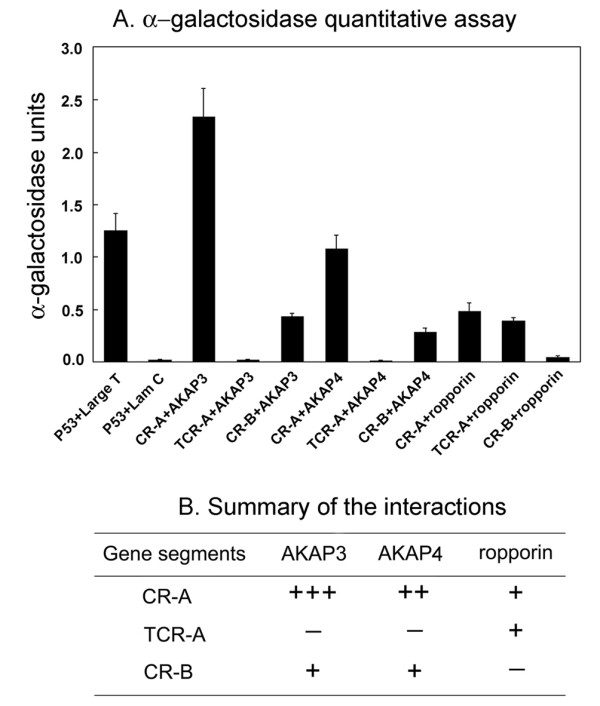
**α-Galactosidase assay and summary of the interactions**. A: α-Galactosidase assay. Quantitative data are plotted with error bars (Standard Deviation) corresponding to three or four separate experiments. The α-galactosidase activity of the transformant bearing CR-A+AKAP3 is much higher than that of P53 and SV40 large T, while the activity of the transformant bearing CR-A+AKAP4 is comparable to that of P53 and SV40 large T. TCR-A+AKAP3 and TCR-A+AKAP4 have very low α-galactosidase activities, similar to the p53+Lam C (negative control). CR-B+AKAP3, CR-B+AKAP4, CR-A+ropporin and TCR-A+ropporin show much lower α-galactosidase activities than P53 and SV40 large T (positive control) but are significantly higher than p53+Lam C (negative control), suggesting that there may be some degree of weak interaction between these pairs of proteins. CR-B+ropporin also show very low α-galactosidase activity similar to the p53+Lam C (negative control). B: Summary of the interactions between segments of CABYR and AKAP3/AKAP4/Ropporin.

These findings were confirmed by quantitation of α-galactosidase activity in the yeast two hybrid systems. The transformant bearing CR-A+AKAP3 had a much higher α-galactosidase activity than that of P53 and SV40 large T (positive control), while CR-A+AKAP4 activity was comparable to the positive control. CR-B+AKAP3, CR-B+AKAP4, CR-A+Ropporin and TCR-A+Ropporin showed activities that were significantly lower than the positive control but notably higher than the negative control. On the other hand, TCR-A+AKAP3, TCR-A+AKAP4 and CR-B+ropporin had low α-galactosidase activities, similar to that of P53 and lam C (negative control) (Fig. 10A). The interactions between CABYR and AKAP3/AKAP4/Ropporin are summarized in Fig. 10B.

The yeast two-hybrid data not only verified the existence of interactions between CABYR and AKAP3/AKAP4/ropporin, but also provided further evidence that the RII like domain of CABYR is critical for its binding with AKAP3/AKA4, even though it does not appear to be requisite for binding with ropporin. The CABYR-B of CABYR does participate in a relatively weak binding with both AKAP3 and AKAP4 but does not bind with ropporin. It is notable that the strengths of the interactions between CABYR-A and both AKAP3 and AKAP4 were much stronger than that of P53 and SV40 large T according to α-galactosidase quantitation.

## Discussion

### Mouse CABYR variants undergo oligomerization

Four splice variants involving two coding regions, CR-A and CR-B, are noted in mouse CABYR [19]. The N-terminus of human CABYR-A contains domains homologous to the RII dimerization and AKAP-binding domains of PKA, suggesting that human CABYR may self-assemble and bind to AKAPs [9]. The RII domains in mouse CABYR, PKA, Sp17, and ropporin share a high degree of identity and similarity with one another as well as with their human orthologues. To test for the formation of mouse CABYR dimers or oligomers, we performed 2-D diagonal electrophoresis [27] in which the first dimension was conducted under non-reducing conditions and the second dimension was subsequently run under reducing conditions. Protein complexes were visualized in the first (non-reducing) dimension, and, after reduction, spots representing the components of complexes that initially contained disulfide (S-S) bonds, appeared in the gel below the diagonal. The combined analysis of diagonal gel immunoblots and non-reducing and reducing 1-D blots clearly demonstrated the oligomerization of mouse CABYR and revealed the composition of individual protein complexes.

At least two different heterodimers of CABYR were found in mouse sperm CABYR. The first, predominant, heterodimer of ~110-130 kDa is comprised of an 80 kDa CABYR-A only component(this isoform has been confirmed to be a CABYR-A only variant and correspond to mCABYR-I by combined the previous work [10,19] and mass spectrometry peptide sequencing (data not shown)) and a 50 kDa form (containing both CABYR-A and CABYR-B). The second, ~70 kDa, heterodimer is composed of a 50 kDa variant that contains both CABYR-A and CABYR-B and a ~20 kDa CABYR form or another protein which binds with CABYR isoforms. Homodimers of CABYR were also possibly existed. A nearly 100 kDa band is likely a homodimer composed of two 50 kDa variants, while a 200 kDa oligomer consists of 80 kDa (CABYR-A only) and 50 kDa variants (containing both CABYR-A and CABYR-B). These observations show that the 80 kDa (CABYR-A only) and 50 kDa variants (CABYR-A and CABYR-B), are the two major forms of CABYR that form homodimers or heterodimers. It is possible that CABYR may oligomerize further through different combination of these isoforms.

### CABYR assembly into the FS in the late steps of mouse spermiogenesis

To characterize the developmental changes in mouse CABYR during spermatogenesis, we conducted a survey of all the stages in the cycle of the mouse seminiferous epithelium in sections of mouse testis by indirect immunofluorescence. Combined with immunogold electron microscopy on mouse sperm in this paper and on human sperm in the reference [9], the changes in localization of CABYR during mouse spermiogenesis suggest that it was most likely incorporated onto the surface of the FS.

Comparison of the expression and localization patterns of mouse CABYR with those reported for other FS proteins [9,30-32] suggests that CABYR is probably incorporated onto the FS surface after several other FS proteins have assembled. AKAPs are major constituents of the FS [33]. AKAP3 expression was first observed in the cytoplasm of round spermatids in step 4 (stage IV) and in the flagellum of elongating spermatids in step 9 (stage IX) [34]. Thus, AKAP3 expression in both cytoplasm and flagellum occurred much earlier than the expression of CABYR. Previous studies showed that the precursors of longitudinal columns appeared earlier than those of the ribs in the rat, column precursors being seen first in round spermatids, and precursors of the ribs making their appearance only in late elongating spermatids [32,35,36]. Since AKAP3 appeared in the ribs and not in the longitudinal columns in human sperm [4], it has been suggested that AKAP3 is incorporated into the FS concurrently with formation of the ribs, but only after formation of the longitudinal columns [34].

In contrast, the temporal pattern of CABYR expression more closely resembles that of AKAP4 expression, which was first detected in both the cytoplasm and flagellum of step 14 (stage II-III) condensing spermatids and disappeared from the cytoplasm of condensing spermatids after step 15 [34]. It has been suggested that the incorporation of AKAP4 into the longitudinal columns and ribs is a major step in completion of assembly of the integrated FS [34] for several reasons. First, AKAP4 is present in both the longitudinal columns and ribs [37] and it constitutes about 40% of the 4 M urea-insoluble components of the FS [38]. Second, targeted disruption of the AKAP4 gene causes defects in the formation of the FS with only nascent longitudinal columns and thin circumferential ribs being present, accompanied by a lack of progressive motility [39]. Third, formation of the definitive FS occurs from distal to proximal during steps 14 and 15 in mice [40] concomitant with the appearance of AKAP4 in that location. By comparison with AKAP4, CABYR appears a little earlier in the cytoplasm but a little later in the flagellum, and its disappearance in the cytoplasm also occurs slightly later than AKAP4. In any event, the present data on the initiation of expression, apparent migration, and final localization of CABYR strongly suggest it is also one of the important constituent proteins of the mouse FS. CABYR may be added to the surface of the FS, representing one of the final steps in formation of this structure during the latter part of spermiogenesis.

### Interaction between CABYR and AKAP3/AKAP4

Bioinformatic analysis indicated that a domain in CABYR-A of both human and mouse CABYR, like ropporin and Sp17, shares a strong sequence similarity with the conserved RIIα domain of PKA [9]. Ropporin and Sp17 showed significant binding with AKAP3 [20,22-24], strongly suggesting that mouse CABYR could also bind with AKAP3 as well as AKAP4. Since AKAPs are highly insoluble proteins [23,33,41], we used a novel immunoprecipitation protocol to identify their potential interaction. These results not only showed that CABYR interacts with both AKAP3 and AKAP4, but also confirmed that the CABYR-A only form can bind with CABYR-B containing variants, thereby forming dimers or oligomers which may be prerequisite for binding to other proteins.

As seen on 2-D immunoblots with antisera specific to CABYR-A and CABYR-B, the four murine CABYR splice variants [19] appear to encode three major clusters of proteins with apparent masses of 80 kDa, 50 kDa, and 28-40 kDa, each of which shows considerable microheterogeneity in charge variants. These protein clusters may correspond to isoforms mCABYR-I (or mCABYR-IV, the two variants express the same protein), mCABYR-III and mCABYR-VI which were previously cloned and sequenced from mouse testis and shown to have deduced masses of 48.3 kDa, 41.3 kDa, and 23.9 kDa. Of these CABYR isoforms the mCABYR-I or mCABYR-IV and mCABYR-III proteins and their charge variants were recovered in AKAP3 and AKAP4 immunoprecipitates, suggesting that isoforms mCABYR-I or mCABYR-IV and mCABYR-III are the major CABYR forms that mediate interactions with AKAPs.

Yeast two-hybrid analyses showed that the interaction between CABYR and AKAP3 is much stronger than the positive control interaction between P53 and SV40 large T, while the strength of the interaction between CABYR and AKAP4 was comparable to that of P53 and SV40 large T. When a truncated CR-A was constructed by deleting the RII domain, CABYR-A losts its capacity to bind with both AKAP3 and AKAP4. This demonstrates for the first time that an interaction between CABYR and the scaffolding protein AKAP3, as well as AKAP4, in the FS of mouse sperm is mediated by the RII-like domain on the N-terminal of CABYR. Interestingly, the co-transformants bearing the CR-B construct and an AKAP3 or AKAP4 construct also grew in the selective medium, albeit more slowly, suggesting that there is some degree of interaction as well between CABYR-B and AKAP3/AKAP4. α-Galactosidase quantitative assays also reflected weak but definite interactions between CABYR-B and AKAP3/AKAP4. Overall, the interaction between AKAP3 and CABYR in this paper could reasonably be speculated to be direct, since it has been demonstrated in vitro [22].

### Interaction between mouse CABYR and ropporin

Mouse ropporin was reported previously [7] to be located mainly on the inner surface of the FS. This is close to that of human CABYR, which is present in the FS, on the surface of the longitudinal column and ribs, and over electron-dense material lying between the FS and the outer dense fibers [9]. Biochemical characterization showed that ropporin, unlike the intrinsic components of the FS, is essentially a soluble protein that binds to the FS under some conditions [7]. Similarly, our observations showed that mouse CABYR, like human CABYR, is present in both Triton X-100 soluble and insoluble fractions, so the soluble form would be expected to be able to bind to the surface of the FS as does ropporin. Furthermore, the N-terminal sequences of both CABYR and ropporin are highly homologous to the dimerization motif of RIIα of protein kinase A.

The similar patterns of localization, biochemical characteristics and molecular structure between CABYR and ropporin suggest that the two proteins have some relationship in the FS. The present study showed that the ropporin can be co-immunoprecipitated with CABYR by anti-CABYR-A polyclonal antibody using the standard co-immunoprecipitation method and that CR-A and ropporin co-transformants grew on medium stringency plates in the yeast two hybrid assay. This observation that CABYR is potentially interacting with ropporin is also bolstered by a paper in which a yeast two-hybrid analysis using SP17 as bait also pulled out ropporin [42]. The binding of CABYR with ropporin appears to require not only the RII domain but also some other domain(s). This speculation is also supported by Hsu et al [43], in which the proline-rich extensin-like domain of CABYR, rather than the RII-like domain, is implicated in mediating the oligimerization of CABYR. Considering the known interactions between AKAPs 3 and 4 [34], AKAP3 and ropporin [20,22], AKAP3 and 4 with CABYR ([22] and in this paper), it is also possible that ropporin is not binding CABYR directly, but is being pulled down in a complex.

## Conclusions

The results revealed three novel aspects of mouse CABYR biology. (1) Mouse CABYR forms homodimers, heterdimers and oligomers mainly involving the most abundant 80 kDa variant (CABYR-A only) and the 48 kDa form (containing CABYR-A and CABYR-B). (2) Mouse CABYR expression begins in the spermatid cytoplasm at step 11 of spermiogenesis and progressively increases though steps 13-15, and CABYR subsequently localizes on the surface of the FS during steps 15-16. (3) Mouse CABYR binds with AKAP3 as well as AKAP4 mainly by its RII-like domain, and has a weak interaction with ropporin that is mediated by a non-RII-like domain or could be mediated by an AKAP scaffold. This is the first evidence that CABYR binds not only with the scaffolding proteins AKAP3 and AKAP4 but also related with ropporin, another RII-like domain-containing protein. These interactions strongly suggest that CABYR participates in the assembly of complexes in the FS, which may be related to calcium signaling.

## Competing interests

The authors declare that they have no competing interests.

## Authors' contributions

YFL conceived of the study, carried out the oligomerization, immunoprecipitation and drafted the manuscript. WH carried out the yeast two-hybrid assay. YHK carried out the gene cloning and antibody production. AM participated in part of the immunoprecipitation study. LD participated in the immunofluorescence study. KK carried out the study by immunoelectron microscopy. CJF participated in the design of the study and helped to draft the manuscript. JCH participated in its design and coordination and helped to draft the manuscript. All authors read and approved the final manuscript.

## Supplementary Material

Additional file 1 **Figure S1**. SDS-PAGE gel electrophoresis of expressed and purified mouse CABYR-A and CABYR-B. Click here for file

Additional file 2 **Figure S2**. Demonstration of the reactivity of the guinea pig antisera with each recombinant form of CABYR. Click here for file
