# Supplementary material for: CABYR isoforms expressed in late steps of spermiogenesis bind with AKAPs and ropporin in mouse sperm fibrous sheath
Source: Reprod Biol Endocrinol. 2010 Aug 23;8:101. doi: 10.1186/1477-7827-8-101 (PMC3398308; doi:10.1186/1477-7827-8-101)
Supplement: Additional file 1 — Figure S1. SDS-PAGE gel electrophoresis of expressed and purified mouse CABYR-A and CABYR-B. [file 1477-7827-8-101-S1.pdf]

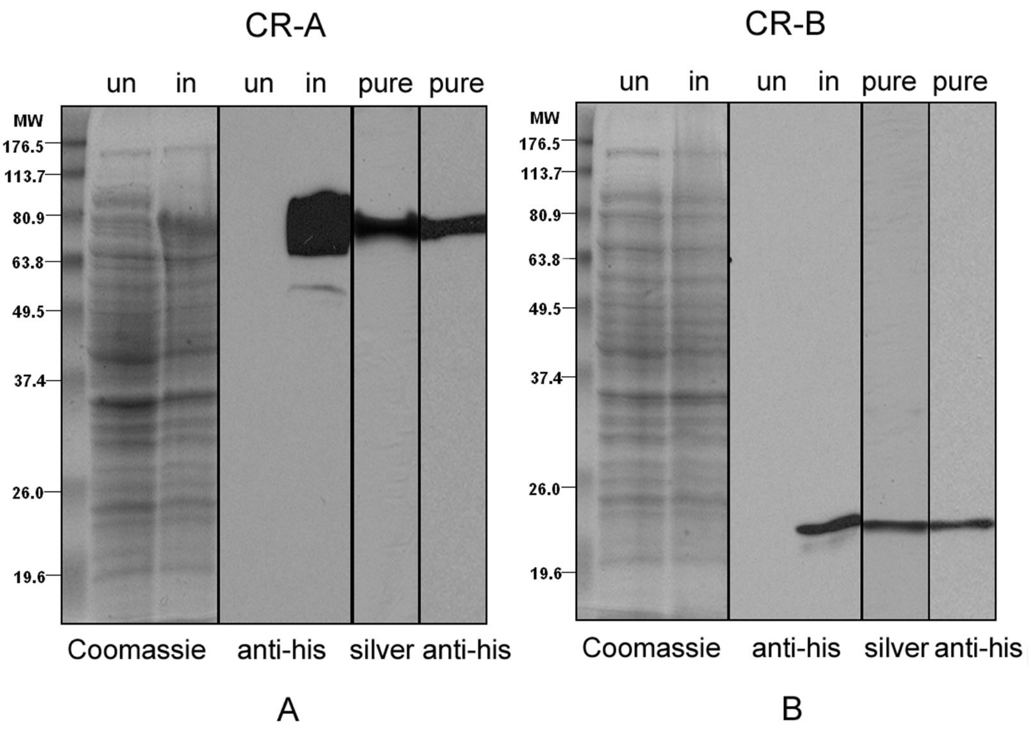

Fig. S1. SDS-PAGE gel electrophoresis of expressed and purified mouse CABYR-A and CABYR-B. CABYR-A (A) and CABYR-B (B) protein bands expressed in BLR (DE3) host cells were observed by Coomassie blue staining in induced lanes (in) and confirmed as recombinant proteins by reaction with anti-His antibody (anti-his). Purity of recombinant proteins (pure) eluted from his-binding columns was examined in the gel by silver staining (silver) and by Western analysis using anti-His antibody.
