# Supplementary material for: CABYR isoforms expressed in late steps of spermiogenesis bind with AKAPs and ropporin in mouse sperm fibrous sheath
Source: Reprod Biol Endocrinol. 2010 Aug 23;8:101. doi: 10.1186/1477-7827-8-101 (PMC3398308; doi:10.1186/1477-7827-8-101)
Supplement: Additional file 2 — Figure S2. Demonstration of the reactivity of the guinea pig antisera with each recombinant form of CABYR. [file 1477-7827-8-101-S2.pdf]

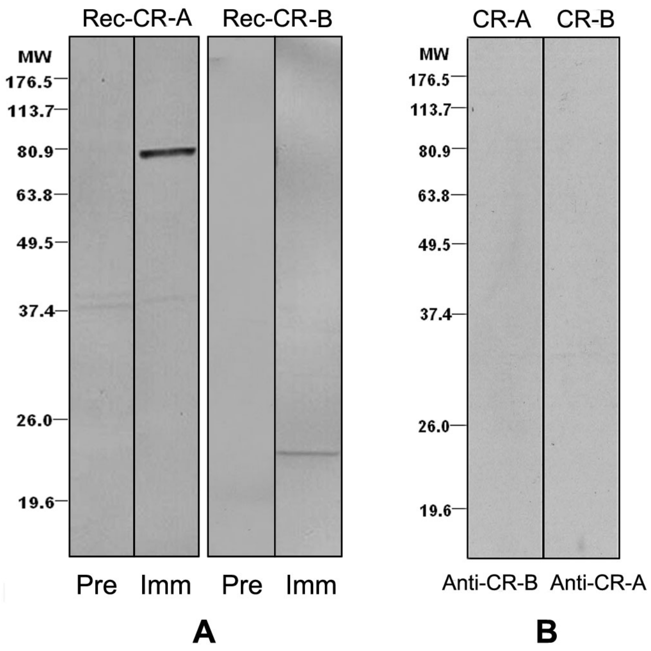

Fig. S2. Demonstration of the reactivity of the guinea pig antisera with each recombinant form of CABYR. The guinea pig anti- CABYR-A and anti-CABYR-B sera recognized the purified recombinant CABYR-A and CABYR-B, respectively, compared with the corresponding pre-immune controls (A). The specificity of each anti-serum was demonstrated further by analysis of cross-reactions by Western blotting (B). Anti-CABYR-B did not react with recombinant CABYR-A and anti-CABYR-A did not react with recombinant CABYR-B.
